# Long-Term Field Data and Climate-Habitat Models Show That Orangutan Persistence Depends on Effective Forest Management and Greenhouse Gas Mitigation

**DOI:** 10.1371/journal.pone.0043846

**Published:** 2012-09-07

**Authors:** Stephen D. Gregory, Barry W. Brook, Benoît Goossens, Marc Ancrenaz, Raymond Alfred, Laurentius N. Ambu, Damien A. Fordham

**Affiliations:** 1 Environment Institute and School of Earth and Environmental Sciences, University of Adelaide, Adelaide, Australia; 2 Organisms and Environment Division, School of Biosciences, Cardiff University, Cardiff, United Kingdom; 3 Danau Girang Field Centre, c/o Sabah Wildlife Department, Kota Kinabalu, Sabah, Malaysia; 4 Hutan/Kinabatangan Orang-utan Conservation Programme, Sandakan, Sabah, Malaysia; 5 North England Zoological Society, Chester Zoo, Chester, United Kingdom; 6 Borneo Conservation Trust, Kota Kinabalu, Sabah, Malaysia; 7 Sabah Wildlife Department, Kota Kinabalu, Sabah, Malaysia; Smithsonian's National Zoological Park, United States of America

## Abstract

**Background:**

Southeast Asian deforestation rates are among the world’s highest and threaten to drive many forest-dependent species to extinction. Climate change is expected to interact with deforestation to amplify this risk. Here we examine whether regional incentives for sustainable forest management will be effective in improving threatened mammal conservation, in isolation and when combined with global climate change mitigation.

**Methodology/Principal Findings:**

Using a long time-series of orangutan nest counts for Sabah (2000–10), Malaysian Borneo, we evaluated the effect of sustainable forest management and climate change scenarios, and their interaction, on orangutan spatial abundance patterns. By linking dynamic land-cover and downscaled global climate model projections, we determine the relative influence of these factors on orangutan spatial abundance and use the resulting statistical models to identify habitat crucial for their long-term conservation. We show that land-cover change the degradation of primary forest had the greatest influence on orangutan population size. Anticipated climate change was predicted to cause reductions in abundance in currently occupied populations due to decreased habitat suitability, but also to promote population growth in western Sabah by increasing the suitability of presently unoccupied regions.

**Conclusions/Significance:**

We find strong quantitative support for the Sabah government’s proposal to implement sustainable forest management in all its forest reserves during the current decade; failure to do so could result in a 40 to 80 per cent regional decline in orangutan abundance by 2100. The Sabah orangutan is just one (albeit iconic) example of a forest-dependent species that stands to benefit from sustainable forest management, which promotes conservation of existing forests.

## Introduction

It has been long recognized that deforestation, conversion of primary or secondary forests to agricultural and other land-use types, is the biggest threat to insular Southeast Asia’s biodiversity [1], [2]. Yet, despite knowledge that deforestation results in population extirpation and even species’ extinction, the rate of deforestation in insular Southeast Asia remains among the highest in the world [3]. The potential for a massive extinction event in Southeast Asia is high [2], especially if habitat loss acts synergistically with other increasingly important extinction drivers, such as climate change [4]. Evaluating the long-term consequences of regional deforestation and its interaction with global climate change in a spatial context is a challenging yet important exercise. Here, we describe a framework to evaluate the relative influences of land-cover and climate change on the future spatial abundance of threatened populations and prioritise individual habitat patches to maximize the probability of their long-term persistence.

The Bornean orangutan *Pongo pygmaeus* is threatened with extinction from habitat loss and degradation (http://iucnredlist.org/apps/redlist/details/17975). Its population has decreased by over 50% in the past 60 years [5], and by over 85% in the last 10,000 years following post-glacial sea-level rise [6]. Recent population estimates range between 45,000–69,000 individuals [7]. Despite growing awareness of the orangutan plight, Southeast Asian deforestation rates continue to increase: the estimated rate of deforestation in Southeast Asia between 1990–97 was 0.91% per annum (pa) [8], whereas a more recent estimate for 2000–10 put it at 2.20% pa [9].

In contrast to the rest of Southeast Asia, the deforestation rate in Sabah (the northernmost Malaysian state on Borneo) has slowed in recent years. Sabah was among the first places to develop industrialized deforestation on Borneo. By the 1980–90s, with the use of heavy machinery, the rate of deforestation peaked at an estimated 1.37% pa [10]. Sabah’s post-1990 deforestation rate has since, however, declined to an average of 0.75% pa [11]. Although encouraging, this rate reduction might be an artefact of forest management; Sabah’s Permanent Forest Estate (some 51% of the state’s land area) is protected from illegal logging while almost all unprotected forest has already been felled [11].

In an attempt to conserve dwindling timber resources, the Sabah Forestry Department has committed to implementing Sustainable Forest Management (SFM) across all their commercial forest reserves. Compared to conventional timber extraction techniques that damage non-target trees and seedlings [12], SFM includes Reduced Impact Logging and selective logging with minimal collateral damage (ITTO: http://www.itto.int). After a successful SFM trial in Deramakot forest reserve initiated in 1997, six major forest reserves are now under SFM, approximately 77.5 km^2^ or 20.2% of Sabah’s Permanent Forest Estate [13]. However, the Sabah government is committed to extending SFM to all of its forest reserves by 2014, such that 51% of the State land cover would constitute good quality secondary regrowth forest.

Reducing Emissions from Deforestation and Forest Degradation (REDD) is a Payment for Environmental Services scheme that attaches financial value to carbon stored in forests, offering incentives for developing countries to reduce emissions from deforestation and forest degradation [14]. Frameworks such as REDD, therefore encourage SFM and could have important benefits for biodiversity conservation (e.g., [15]), including orangutan conservation.

With an estimated 11,000 individuals, Sabah is considered the stronghold for the Bornean orangutan subspecies *P. p. morio* [16]. In this study, we develop a framework to examine the consequences of future SFM implementation scenarios on the long-term persistence of Sabah’s orangutan population. However, rather than considering forest management in isolation, we recognize that the effects of habitat loss will likely be exacerbated by global climate change [4] and incorporate this synergy in our model. There is large uncertainty in climate change projections for the tropics and their consequences for tropical biodiversity [17]. To our knowledge, there have been no studies of orangutan climate preferences or tolerances, although by comparison to the other orangutan species and subspecies, it would appear more drought-tolerant. Climate could, however, be an important determinant of their fundamental niche if, for instance, ENSO-induced droughts or fires limit the availability of preferred and “fallback” foods, including barks and leaves [18].

To examine how forest management and climate change might affect the Sabah orangutan population, we modelled their distribution and abundance using a Species Distribution Model (SDM) and projected it onto regional land-cover and global climate change projections. Our framework improves on bioclimate-envelope models by linking them to dynamic land-cover projections, allowing us to evaluate the relative influence of climate and land-cover change in a scenario analysis. Due to its spatially-explicit nature, this framework allows managers to evaluate the current-day and longer-term importance of individual habitat patches for population persistence. We predicted that the Sabah orangutan population would fare best under the scenario characterising minimum land-cover change and maximum climate change mitigation, hypothesising that land-cover change will further degrade primary forest and that climate – particularly temperature – in currently occupied habitat will change, rendering the habitat less suitable. We also predicted that land-cover change would have a greater influence on orangutan population abundance than climate change because the rate of change in the later is currently faster than the latter, but that their combined impact would be higher than when considered in isolation.

## Methods

### Nest Count Data

**Figure 1:**
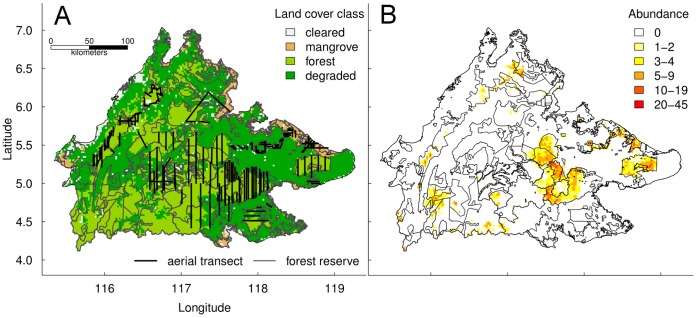
Observed and predicted orangutan nest distributions. Maps showing (a) the distribution of orangutan nest counts across Sabah in all survey years and (b) the hurdle Species Distribution Model predictions for the present day.

To monitor major *P. p. morio* populations across Sabah, the Kinabatangan Orangutan Conservation Project has counted orangutan nests along approximately 3366 km of aerial transects through 19 forest reserves and parks over six years (2001–3, 2007 and 2009–10) (Figure 1a). Details of the aerial nest count protocol can be found in [16]. Two details with potential to bias our results are: (1) surveys were along transect that were occasionally repeat-surveyed introducing spatiotemporal autocorrelation that might cause abundance overestimates, and (2) surveys were mostly flown over forest reserves that were considered prime orangutan habitat and so might overestimate their distribution.

To guard against spatiotemporal autocorrelation we calculated average spatiotemporal orangutan habitat suitability. To reduce any influence of spatial autocorrelation, we aggregated counts to 2.5 km^2^ grid cells, chosen because it is the average female orangutan territory size [19] and showed weaker spatial autocorrelation than at a finer 1 km^2^ resolution (mean Local Moran’s *I*: 1 km  = 0.580, 2.5 km  = 0.540, range  =  −1 to +1). To reduce temporal autocorrelation between repeat surveys, we used the average nest count in each cell calculated over the entire monitoring period. The final dataset had 1180 gridded aerial nest count cells. All spatial data processing was done in *R* (www.r-project.org) using packages *raster* and *sp*.

Failing to survey over-degraded and unforested areas with few or no nests introduces a systematic survey bias that will prevent a statistical model from distinguishing those areas as unsuitable. Consequently, we selected a number of unsurveyed point locations to represent “pseudoabsences”. These were selected at random from outside forest reserves, to distinguish them from “true absences” (i.e., a zero nest count in a forest reserve). To counter uncertainty in whether the pseudoabsences (or, indeed, observed absences) were true absences, we used a statistical threshold to identify model predicted presences that down-weights the influence of pseudoabsences (the Maximum Sum of Specificity and Sensitivity threshold; [20]). Our data therefore represented two processes: (1) information on whether a species is present, and (2) information on the species’ abundance in areas where it is present.

### Predictors of Nest Abundance

We identified a number of variables considered important predictors of orangutan presence and abundance, and collated spatial data to represent them (Table 1). We processed them to match the nest count data resolution (Table S1). The predictors were uncorrelated for cell values at the locations of nest counts and for all cells across Sabah (all Spearman correlations ≤0.68), except elevation and slope (Spearman *r* = 0.76). We chose to retain both elevation and slope because orangutan prefer lowland forests (elevation) and because we expected forest in difficult terrain to remain standing (slope) (Table 1). We classified the predictors into three classes: (i) *Anthropogenic* activities were expected to exile orangutan from otherwise suitable habitat, (ii) *Habitat* variables were expected to influence orangutan densities, and (iii) *Climate* variables were expected to delineate orangutan preferred average seasonal climatic conditions.

**Table 1:** Spatial predictors used to build the Species Distribution Model with notes on their perceived importance for orangutan and data source.

Name	Class	Description	Relation to orangutan	Source
popdist	Anthropogenic	distance to a major population centre(250000+ people)	population centres are unsuitable habitat and a source of disturbance	SWD
roaddist	Anthropogenic	distance to a main road	roads increase mortality and reduce dispersal	SFD
riverdist	Habitat	distance to a river	rivers are used as dispersal routes and provide native riverine vegetation	SFD
protectarea	Habitat	areas in which logging is prohibited	protected areas will be vital for long-term orangutan persistence	SWD & SFD
elevation	Habitat	meters above sea level	orangutans prefer habitats at lower altitudes	SRTM
slope	Habitat	degrees of inclination from the horizontal	steep slopes are difficult to develop and might provide refuge	SRTM
forest	Habitat	2009–10 forest cover	includes the forest reserves and unprotected forest	CRISP
degraded	Habitat	2009–10 degraded cover	severely degraded vegetation areas including small-scale plantations	CRISP
mangrove	Habitat	2009–10 mangrove cover	considered suboptimal orangutan habitat butless prone to development	CRISP
climate	Climate	mean 1989–2009 annual temperature and monthly wet dry season rainfall	included to quantify orangutan climatic tolerances	CRU TS v3

### Species Distribution Modelling

Over 43% of the aggregated orangutan nest counts were zero, and this increased to 73% when pseudoabsences were included. We modelled these zero-augmented data as originating from a hurdle process characterizing two separate processes: (1) presence across the landscape depending on whether or not habitat is suitable, and (2) abundance in suitable habitat. We assumed a hurdle approach because it has been shown to outperform alternatives [21]. A hurdle model is a two part, mixed model that predict probability of presence (and absence) and then abundance contingent on presence. This allowed us to identify factors influencing presence and abundance separately; an important benefit when examining the spatial abundance of rare or declining species.

We built a boosted regression tree SDM because they readily fit non-linear and interacting processes and have been used to model hurdle processes [22]. We fitted a saturated hurdle model using fitting parameters: learning rate (*lr*) = 0.01, bag fraction (*bf*) = 0.7, first-order interactions (*tc*) = 2 and number of trees in the range *nt* 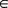
[10, 35, …, 15000]. In essence, parameters *lr*, *bf* and *nt* control the contribution of each term to the regression model, the proportion of data randomly selected to build the regression model and the number of regression terms, respectively. To determine the optimum *nt*, we compared the out-of-sample predictive performance of models fitted with increasing *nt* where predictive performance was calculated as mean prediction deviance over 10 cross-validation subsets (D*cv*). We used the model with *nt* that minimized D*cv* [23]. Models were fitted in *R* using package *gbm* and *dismo*.

To optimize the predictive performance of the saturated hurdle model, we fitted the binomial and Poisson parts separately using all possible fitting parameter combinations in the space defined by *lr* 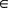
[0.01, 0.005, 0.001], *tc* 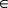 [1], [2], [3] and *bf* = 0.7. Again, we varied *nt* 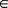
[10, 35, …, 15000]. The *optimized* model was that with the combination of fitting parameters that minimized D*cv*. Finally, we compared the optimized saturated model to models simplified by removing the least influential predictors until the change in D*cv* exceeded the original cross-validation prediction deviance SE, repeating this for the binomial and Poisson parts separately and together [23].

We calculated model explanatory performance for the Binomial part of the hurdle model using confusion matrix-based statistics Kappa and AUC, and mean square error (*mse*) and relative mean prediction error (*rmpe*) for the Poisson part [24].

### Model Validation

We built the SDM using all available nest count survey data and so had no unused data with which to validate its predictions. Instead, we validated our model by examining the relationship between our nest count predictions and an independent aerial nest count data set [25] collected in 2007–10 along transects spaced 3 km apart (*c.f.* 5 km for the main dataset) using GLM and Iterative Re-weighted Least Squares in *R* package *MASS*.

### Scenario Testing

We converted nest count predictions to abundances for scenario testing. The relationship between the number of nests per km of aerial transect (*Ai*) and orangutan density (*Dou*) is given by *Dou*  =  exp(4.730+ (0.980 × ln(*Ai*)))/(*p*×*r*×*t*), where *p* = 0.9 is the proportion of nest building individuals, *r* = 1.084 is the daily rate of nest production and *t* = 286.3 days is the nest decay rate [16].

We developed a spatially-explicit land-cover change model that projected observed regional 2000–10 deforestation and forest regeneration rates to 2100 under the SFM scenarios described in Table 2. We calculated and projected these rates for Sabah forest reserves and unprotected forest separately. Deforestation in forest reserves represented forest degradation due to timber harvesting and was modelled as a constant harvest, independent of remaining forest. Forest reserves were allowed to regenerate after ≈60 years [26]. Deforestation in unprotected forest represented the permanent conversion of forest to degraded or cleared land and was modelled as an annual transition matrix projected as a discrete transition Markov Chain [27]. We calculated deforestation rates from 2000–10 observed land-cover raster layers [28] restricted to areas outside nationally and internationally designated protected areas in which any extraction is strictly prohibited.

**Table 2:** Future land-cover and climate change scenarios evaluated for their effect on orangutan spatial abundance in Sabah.

Scenario	Description	Justification
No Intervention	Sustainable Forest Management (SFM) is implemented only in current SFMforest reserves others are converted to degraded sequentially and regenerateafter 60 years. CO_2_ emissions continue to increase under a no-climate-policyscenario and climate changes unabated	Current SFM is adequate to safeguard theorangutan population, which will not beaffected by climate change
Habitat Intervention	SFM is implemented in all forest reserves but CO_2_ emissions continue toincrease under a no-climate-policy scenario and climate changes unabated	Safeguarding the orangutan populationrequires complete SFM implementationeven under no climate change
Climate Intervention	SFM is implemented only in current SFM forest reserves but CO_2_ emissionsstabilize at 450 ppm by 2100 under a stabilization-policy scenario andclimate change slows	Current SFM is adequate to safeguard theorangutan population but only if climatechange can be slowed
Combined Intervention	SFM is implemented in all forest reserves the Sabah Forest Departmentplans to implement this scenario by 2014. CO_2_ emissions are cut andstabilize at 450 ppm under a stabilization policy scenario andclimate change slows	Safeguarding the orangutan populationrequires complete SFM implementationas climate change affects habitat suit

To identify which raster cells would be changed at each time step and to which class they would change, we used 2010 land-cover prediction probabilities from random forest models built using spatial predictors of land-cover change (Table S2; [29]). Random forest models assign each raster cell a *probability of class membership* to each land-cover class calculated as the proportion of iterations in which they were assigned membership to that class. A cell’s predicted 2010 land-cover class is that which has the highest probability of class membership. We calculated each cell’s *vulnerability to change* as the maximum probability of membership to any other land-cover class [30]. For each time step, the land-cover change model calculated how many and which raster cells to change from the deforestation rate projections and cell vulnerabilities, and changed their land-cover class to that with the second highest probability of class membership. Forest reserves were deforested in sequence, as a decreasing function of the vulnerability of their constituent cells.

Both SFM scenarios were tested under two contrasting global climate change scenarios: (1) a no-climate-policy reference scenario (MiniCAM Ref.; hereafter ‘Ref’), and (2) a corresponding policy (stabilization) scenario (MiniCAM, Level 1; hereafter ‘Pol’) [31]. MAGICC/SCENGEN v.5.3 (http://www.cgd.ucar.edu/cas/wigley/magicc), a coupled gas-cycle/aerosol/climate model, was used to generate the climate anomalies that were an ensemble of seven GCMs chosen on the basis of their skill in reproducing seasonal rainfall (1980–1999) at global and regional (Southeast Asia) scales [32]. The GCMs selected were: BCCRBCM2, CCCMA-31, CSIR0-30, GFDLCM20, MIROCMED, CCSM-30 and UKHADGEM.

## Results

### Orangutan Distribution and Abundance

The optimized saturated hurdle model explained 68% of the deviance in aerial orangutan nest counts and had an overall relative mean prediction error (*rmpe*) of 24% (Table 3). The Poisson part had a higher deviance explained (70.0%) than the binomial part (45.5%) and required second-order interactions (i.e., *tc* = 3). The explanatory power of the binomial part was robust (Kappa = 0.639 calculated using a mean threshold over five methods; AUC = 0.917), as was the Poisson part (mean square error [*mse*] = 4.504, *rmpe* = 20.7%). Model residuals were largest where the model estimated a nest count (Figure S1) and their spatial autocorrelation was low (Global Moran’s *I* = 0.129, range  =  −1 to +1).

**Table 3:** Results of the hurdle boosted regression tree simplification procedure.

Model simplification	Binomial		Poisson		Hurdle			
	D*cv*	SE D*cv*	D*cv*	SE D*cv*	D*null*	D*resid*	*mse*	*rmpe*
saturated	0.696	0.021	3.512	0.272	2.429	0.787	2.417	0.240
binomial	0.944	0.014	3.442	0.435	2.661	0.870	3.040	0.302
Poisson	0.695	0.016	3.822	0.348	2.361	0.896	3.087	0.307
binomial and Poisson	0.946	0.012	3.880	0.304	2.668	1.106	3.866	0.384

Hurdle models were fitted as a two-step process: a binomial and Poisson part. These results show that the saturated model using all spatial predictors for both binomial and Poisson parts had lower prediction deviance (e.g., Dcv) and explanatory deviance (e.g., *mse*) compared to hurdle models for which the binomial, Poisson or both parts were built using only the most influential spatial predictors.

Abbreviations: D*cv* and SE D*cv* are the mean and standard error of the 10-fold cross-validation residual deviances, D*null* and D*resid* are the mean null and residual deviances, *mse* is the mean square error and *rmpe* is the relative mean prediction error.

**Figure 2:**
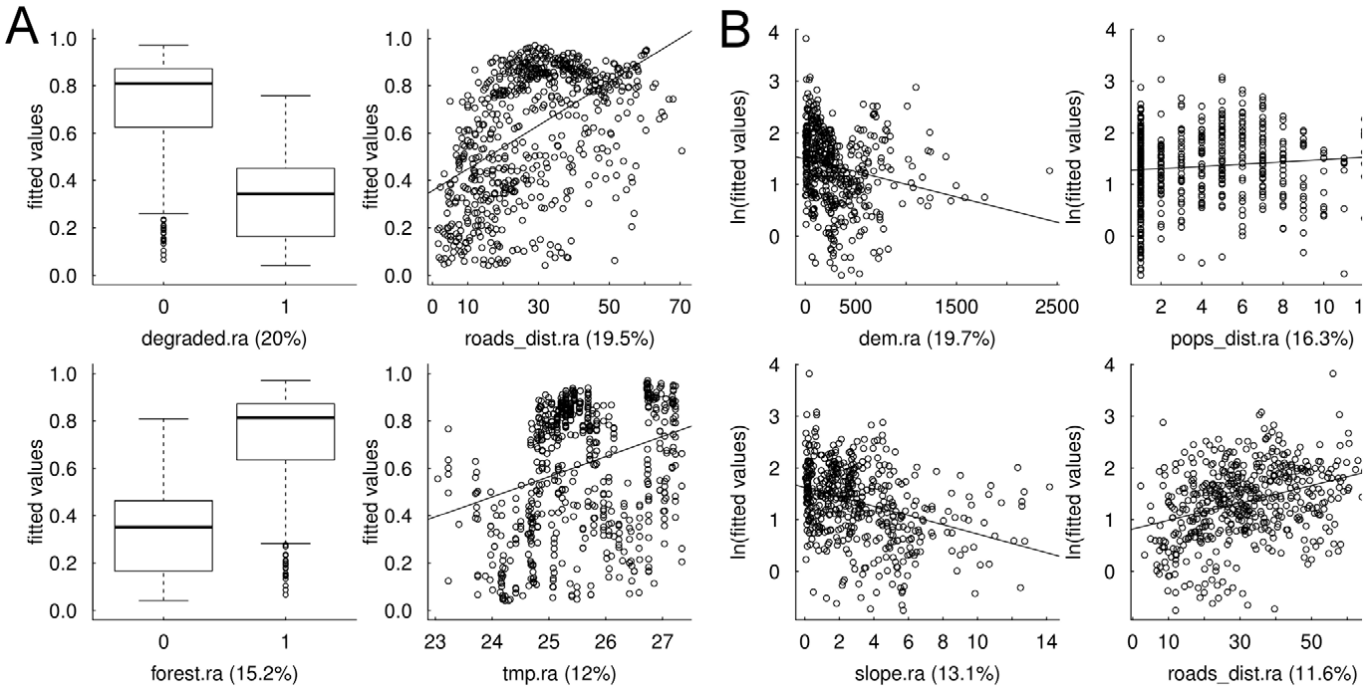
Relationships between nest presence and abundance and their four most influential predictors. Figures showing the relationship between (a) orangutan nest presence and (b) orangutan nest abundance and their four most influential predictors in the final saturated hurdle Species Distribution Model. Solid lines are the robust linear regression fit using Huber weights and fitted by Iterated Re-weighted Least Squares and indicate the direction of the relationship between the response and predictor variables. The relative importance of each predictor is given in parentheses on the *x*-axis. Note: abundance fitted values are ln transformed and zero values are not presented.

The model correctly predicted that the current-day orangutan population is restricted largely to forested areas in eastern Sabah, primarily in Kinabatangan Wildlife Sanctuary, Malua Biobank, Kulamba, Ulu Segama and Deramakot forest reserves and Tabin Wildlife Reserve (Figure 1b). The optimized saturated hurdle model could not be simplified without loss of predictive and explanatory power (Table 3), suggesting that current-day orangutan distribution and abundance is affected by climate, habitat and anthropogenic factors. Orangutan nest presence was most likely in forest further from major roads that were warmer and less disturbed (Figure 2a). Orangutan nests were most abundant at low elevations further from population settlements and major roads where the slope was shallow (Figure 2b). Distance from rivers, mangrove habitat and whether forest was protected or not were scarcely important (Figure 2).

### Model Validation

**Figure 3:**
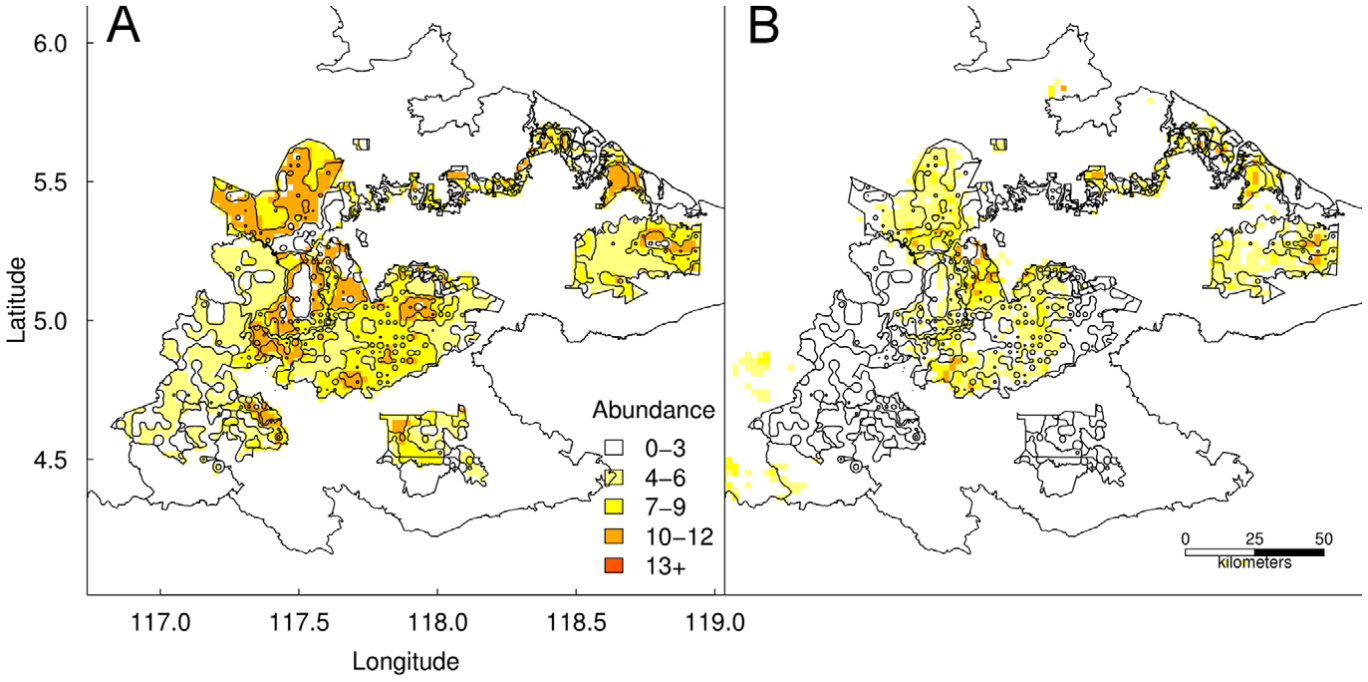
Validation of predicted nest counts on an independent orangutan nest count dataset. Maps showing the spatial correspondence between orangutan nest counts calculated from (a) [25] and (b) our model predictions. Note that the model-predicted nest counts are generally lower than the empirically derived estimates.

Our model validated well, as evident by a strong positive linear relationship with independent data [25] (OLS fit: *y* = 0.288+0.311*x*, IWLS fit: *y*  =  −0.03+0.309*x*; Figures 3 and S2).

### Scenario Testing

The 2000–10 annual deforestation rate in Sabah was 1.18% pa. The rate in unprotected forest (1.26) was higher than in forest reserves (1.13) (Figure S3a). These differences were not, however, reflected in the current-day SFM implementation land-cover change projections because all unprotected forest was deforested within 10 years, while some forest remained in non-SFM forest reserves until 2085 (Figure S3b). In contrast, complete implementation of SFM in all forest reserves resulted in quite different deforestation patterns because forest was not degraded (Figure S4). Details of the importance of spatial predictors and land-cover classification errors are given in supplementary material (Figure S5, Table S3).

By 2100, Sabah mean annual temperature was forecast to increase by 2.5 and 1.2°C under the Ref and Pol scenarios respectively. In contrast, mean seasonal precipitation (wet season: October to March; dry season: April to September) was forecast to remain relatively stable under both scenarios. A notable weakness in projecting SDM is their uncertainty when extrapolating to unobserved conditions. Our precipitation forecasts were always within the observed current-day range (Figures S6 and S7) but mean temperature exceeded maximum observed temperature by 2060. By 2100, the Ref temperature forecast for eastern Sabah was up to 2.2°C above the maximum observed temperature (this was less than 0.8°C under the Pol scenario; Figure S8).

**Figure 4:**
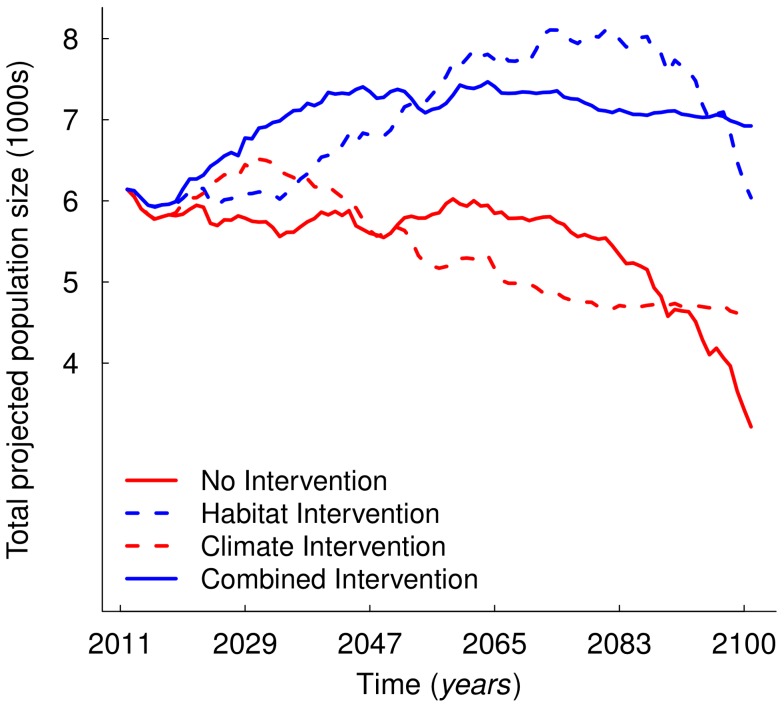
Orangutan population projections under the four intervention scenarios. Time series of total Sabah orangutan population projections under intervention scenarios described in Table 2.

Sabah orangutan population projections under the different SFM and climate change scenarios are shown in Figure 4. The population was projected to decrease by 82 and 36% by 2100 under the “No Intervention” and “Climate Intervention” scenarios but grow by 10% under the “Combined Intervention” scenario. This projected increase was due largely to SFM implementation, as evidenced by the similar Combined and “Habitat Intervention” trajectories, although the population was projected to increase by 22% by 2070 under the latter – the explanation for this appears to be in the spatial pattern of abundance through time.

**Figure 5:**
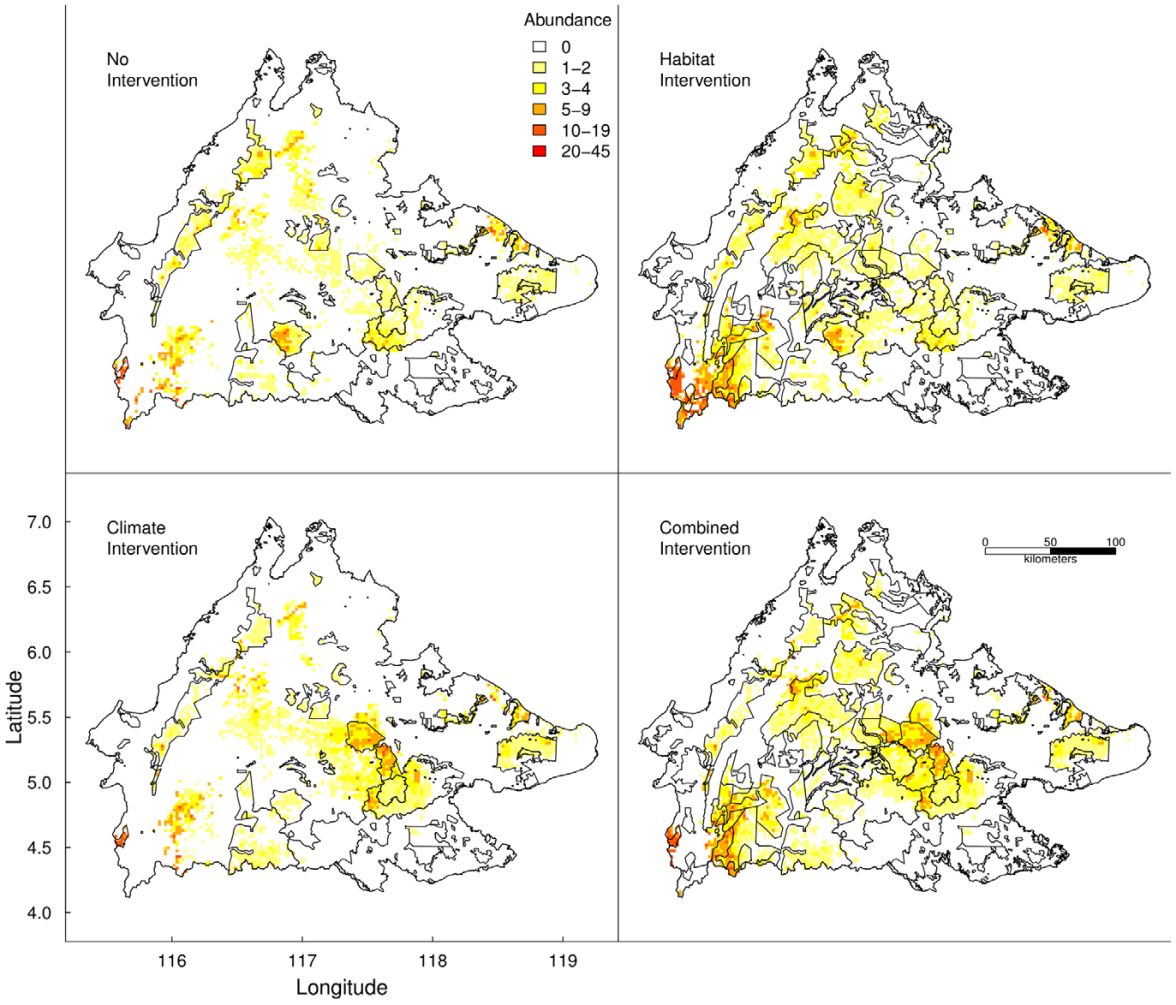
Spatial orangutan abundance in 2100 under the four intervention scenarios. Maps showing 2100 abundance projections for each intervention scenario described in Table 2. The polygons are the sustainably managed forest reserves under each scenario.

Compared to the 2010 spatial abundance pattern (Figure 1b), the orangutan population generally moved west by 2100 across all tested scenarios (Figure 5). In contrast to the Climate and No Intervention scenarios, forest in the west remained standing under the Combined and Habitat Intervention scenarios and was colonised by orangutan. In contrast to the Combined and Climate Intervention scenarios, temperature in the eastern forests increased beyond current-day extremes under the Habitat and No Intervention scenarios and orangutan populations in these forests declined.

## Discussion

By linking regional dynamic land-cover and global climate change models, we were able to evaluate the relative influence of land-cover and climate change on the future spatial abundance patterns of a threatened tropical species and identify potentially important habitat for its long-term persistence. We show that only complete implementation of SFM across all forest reserves (Combined and Habitat Intervention scenarios) will ensure Sabah’s orangutan population does not decline in the long term, and that the population will fare best under a climate change mitigation scenario. Spatially, our model predicted that eastern forest reserves currently support highest orangutan numbers but that, even if they remain forested under complete SFM implementation, their suitability for orangutan will gradually decline as temperature increases under Ref, and to a lesser extent Pol, climate change scenarios. Instead, currently unoccupied western forests might become a future orangutan climate refuge.

If Sabah honours its commitment to implement SFM in all its forest reserves by 2014 [13], then our modelling predicts a positive outlook for the orangutan, with a potential increase in population size by up to 22%, and likely ‘umbrella’ benefits to other species [33] and ecosystem services [34]. If, however, these pledges are not met, then Sabah’s orangutan population is forecast to decline substantially by 2100. For instance, failing to implement SFM in all forest reserves might cause an 80% decline in the orangutan population by 2100 (compare Habitat and No Intervention, Figure 4). Our findings show that climate change and its interaction with habitat loss are likely to be important to orangutan conservation; an important insight beyond existing orangutan population and habitat viability assessments in Borneo [35] and Sumatra [36]. Failure to stabilize global CO_2_ emissions at around 450 ppm might cause the orangutan population to decline by an additional 46% by 2100 (compare Climate and No Intervention, Figure 4). If SFM were implemented in all forest reserves, then the effect of climate change would be felt mostly in eastern Sabah, which will become hotter and less hospitable approaching 2100 (compare Combined and Habitat Intervention, Figure 4).

Our model predicts a gradual re-population of forest reserves in western Sabah where populations have been extirpated by hunting ([37]; Figure 5). Under the Climate and No Intervention scenarios, these areas become heavily degraded and unsuitable for orangutan. If, however, these areas remain forested under SFM, then they might become important under climate change. Compared to the current-day, eastern Sabah is forecast to experience hotter conditions by 2100 but the west will remain climatically suitable (Figure S8). There have been no studies of orangutan climate preferences, tolerances or its effect on their abundance. Our model suggests that climate, specifically mean annual temperature and wet season precipitation, are important determinants of orangutan abundance. If our model captures the orangutan preferred climate niche, and can be extrapolated to novel climates (that arise in eastern Sabah after 2060 for the Ref scenario), then western forests might become a future orangutan climate refuge, underscoring the need to protect them despite their current lack of orangutan.

The validity of our forecasts rests on the assumption that our hurdle model captured the spatial pattern of orangutan distribution and abundance and correctly identified their main drivers. Our SDM was a good descriptor of the highly variable aerial nest count data, explaining almost 70% of their variation, and its predictions embodied SWD wildlife warden on-ground knowledge of orangutan distribution in Sabah. It identified drivers of orangutan presence and abundance supported by expert opinion and the literature. For example, it is well-documented that roads promote deforestation [37] and that orangutan are forest-dwelling animals [38] and our hurdle model predicted highest orangutan presence and abundance in forested areas away from major roads [39]. Although habitat loss is the major threat to orangutan persistence on Borneo, it is closely followed by hunting and illegal trade [37]. Our results indicate that orangutan nests were most abundant away from large human settlements (>250,000 people) suggesting that hunting has left its legacy in orangutan distribution and abundance in Sabah.

There is a growing body of evidence suggesting that orangutan can occupy degraded, even agricultural, landscapes, albeit at lower numbers (e.g., [40]). For example, orangutan have been found living in *Acacia* spp. plantations harvested for paper and pulp [41]. Our model predicted low-density sparse orangutan “patches” outside sustainably managed forest reserves. Only a few small and isolated populations were forecast to persist by 2100 and this seems realistic given reported extirpation of population groups in repeatedly and extensively logged forest in Malua BioBank [42].

Linking dynamic land-cover and climate change projections has been attempted rarely (but see [43]). The concern is that the added uncertainty from the land-cover models will render projections unreliable [44]. By employing a scenario-based analysis our framework will allow – at least – qualitative comparison of feasible management scenarios. Whether static models, such as SDM, are the best modelling approach for such analyses is questionable. There is growing concern that SDM yield unreliable estimates of a species’ fundamental niche and future abundance because they omit highly influential factors, such as harvest and dispersal [45]. Our SDM is not immune to these drawbacks. For instance, it does not incorporate past or future hunting pressure, calling in to question the nest predictions in western forest reserves where orangutan have been extirpated by hunting [37] and assuming that hunting pressure will remain negligible into the future. Although currently inconceivable, even a small increase in hunting pressure could cause a long-term decline in the population (e.g., [36]).

Importantly, our model assumes that dispersal is no constraint on patch colonization. All patches, whether large and connected or small and isolated, have an equal probability of being colonised. In reality many patches will be unreachable by dispersing individuals because they are too far or too isolated [46]. Regarding long-term movements, our SDM was constrained to project orangutan abundance within the political boundary of Sabah. In reality, political boundaries are inconsequential for orangutan that can move through contiguous forest linking countries. Brunei, located to the south west of Sabah, retains a high proportion of standing primary forest currently unoccupied by orangutan. If our model projections prove accurate, then orangutan could migrate into Brunei, particularly if climate change continues unabated. On the other hand, orangutan could migrate south into East Kalimantan, Indonesian Borneo, where hunting pressure remains high and populations would likely be extirpated [47].

To incorporate the effects of extraneous factors, such as hunting and fires, and dispersal and migration into population dynamics and – ultimately – orangutan abundance projections, we recommend that future work focuses on extending these SDMs to a stochastic coupled niche-population modelling such as has already been suggested [48].

The Bornean orangutan population has declined >50% over the past 60 years due to habitat loss and fragmentation. Further decline might be avoided with REDD+ support [15]; we show that complete implementation of SFM across Sabah’s forest reserves will sustain the orangutan population in the long-term without substantial decline. Sabah, however, constitutes only a small fraction of the total orangutan distribution on Borneo, and illegal deforestation and conventional timber logging outside Sabah is widespread. For example, over 56% of lowland forest in Kalimantan was lost between 1985–2001 [49]. To safeguard the Bornean orangutan from extinction (as opposed to local extirpation), we encourage the other countries and provinces on Borneo to follow Sabah’s lead and seek REDD+ support to implement SFM across their timber-producing forest reserves and reconnect fragmented and isolated forest fragments.

### Conclusion

This study provides a framework with which wildlife population managers, including those involved with orangutan conservation on Borneo and Sumatra, can evaluate the relative influence of future regional land-cover and global climate change on the spatial abundance patterns of threatened species and identify regions – even habitat patches – that should be conserved to maximize the probability of their persistence. This framework could help countries justify applications to payment for environmental services schemes, such as REDD and REDD+.

## Supporting Information

Figure S1 **Predicted nest count residuals.** Map of Sabah showing the residuals from the hurdle boosted regression tree Species Distribution Model. Note that the residuals are highest were the model predicted a nest count and lowest were the model predicted a nest presence. Shaded areas are commercials forest reserves and protected areas.
(TIFF)

Figure S2 **Relationship between nest count predictions and an independent orangutan nest count dataset.** Plot showing the ordinary least squares (OLS) and robust iterative re-weighted least squares (IWLS) fits when predicting our SDM model predictions with data published in [25].
(TIFF)

Figure S3 **Land-cover class gains and losses and projected changes between 2010–2100.** Plots showing (a) the observed gains and losses of cells in each land cover class, and (b) the projected changes in each land class between 2010–2100 in forest reserves and unprotected forests for the current-day SFM scenario. Absolute gains and losses in least-widespread classes were negligible compared to changes in degraded land and forest cover. Consequently, projected changes in these land-cover class were inconsequential compared to projected gains and losses in degraded and forest land cover, respectively.
(TIFF)

Figure S4 **Land-cover change projections.** Maps showing land cover change projections at 2011, 2041, 2071 and 2100 under (a) current-day SFM and (b) complete SFM implementations.
(TIFF)

Figure S5 **Predictor importance for predicting 2010 observed land-cover.** Plots showing the relative importance of spatial predictors in predicting observed 2010 land cover in (a) forest reserves and (b) unprotected forests.
(TIFF)

Figure S6 **Per cent wet season precipitation delta maps under each CO_2_ mitigation scenario.** Per cent wet season precipitation delta maps at 2041, 2071 and 2100 relative to 2010 for Ref and Pol scenarios.
(TIFF)

Figure S7 **Per cent dry season precipitation delta maps under each CO_2_ mitigation scenario.** Per cent dry season precipitation delta maps at 2041, 2071 and 2100 relative to 2010 for Ref and Pol scenarios.
(TIFF)

Figure S8 **Degree Centigrade temperature delta maps under each CO_2_ mitigation scenario.** Degrees Centigrade temperature delta maps at 2041, 2071 and 2100 relative to 2010 for Ref and Pol scenarios.
(TIFF)

Table S1 **Spatial predictors.** Spatial predictors used to build the Species Distribution Models and notes on their processing.
(DOC)

Table S2 **Land cover change predictors.** Table of variables considered important predictors of land cover change.
(DOC)

Table S3 **Accuracy statistics for the random forest models of 2010 land-cover predictions.** Prediction error rates were low for the most-widespread land-cover classes but high for the least-widespread classes (e.g., mangrove and cleared land) because they constituted less than 5% of the land cover.
(DOC)
