# Supplementary figures and images for: Long-Term Field Data and Climate-Habitat Models Show That Orangutan Persistence Depends on Effective Forest Management and Greenhouse Gas Mitigation
Source: PLoS One. 2012 Sep 7;7(9):e43846. doi: 10.1371/journal.pone.0043846 (PMC3436794; doi:10.1371/journal.pone.0043846)

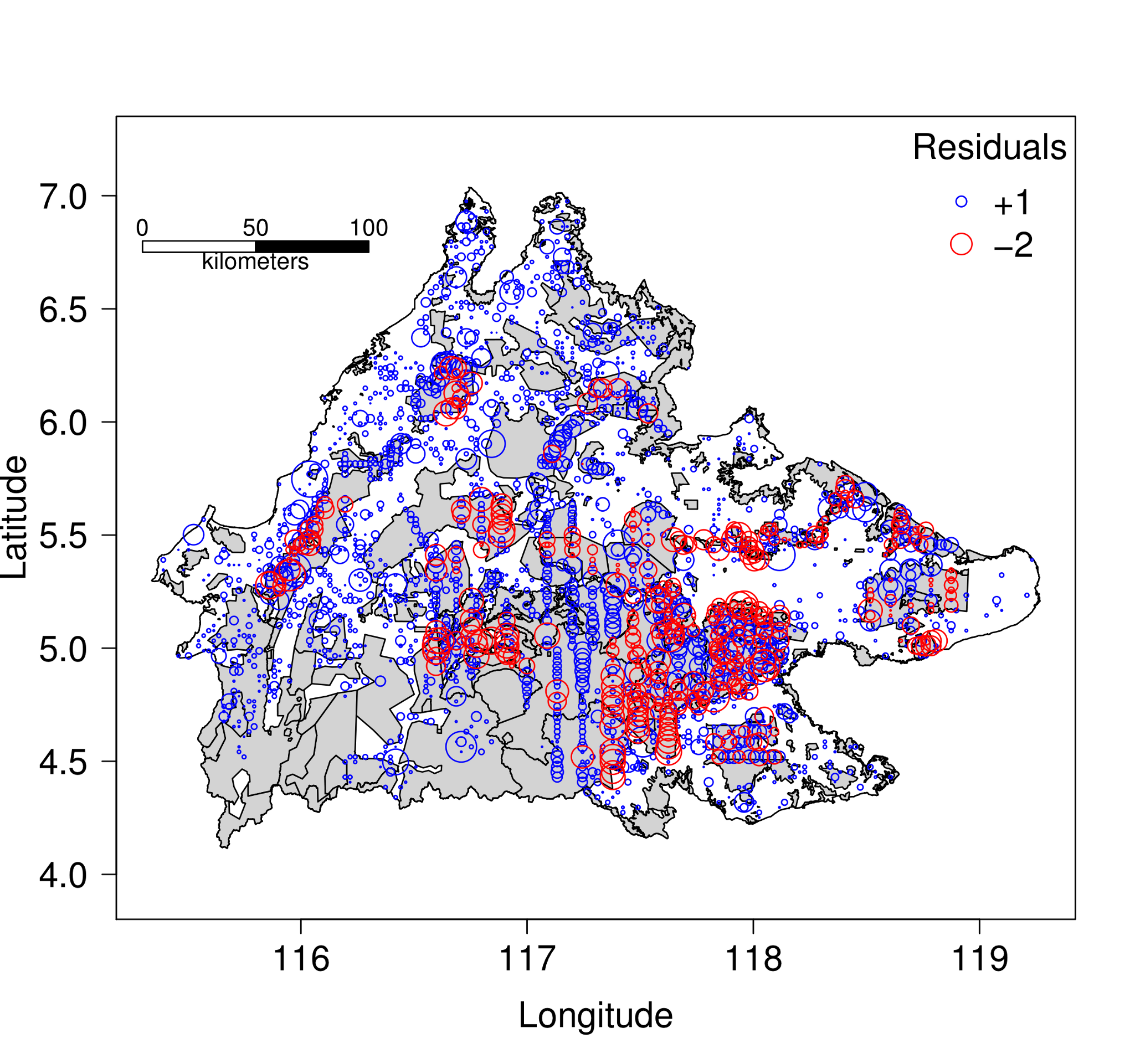

Supplement: Figure S1 — Predicted nest count residuals. Map of Sabah showing the residuals from the hurdle boosted regression tree Species Distribution Model. Note that the residuals are highest were the model predicted a nest count and lowest were the model predicted a nest presence. Shaded areas are commercials forest reserves and protected areas. (TIFF) [file pone.0043846.s001.tiff]

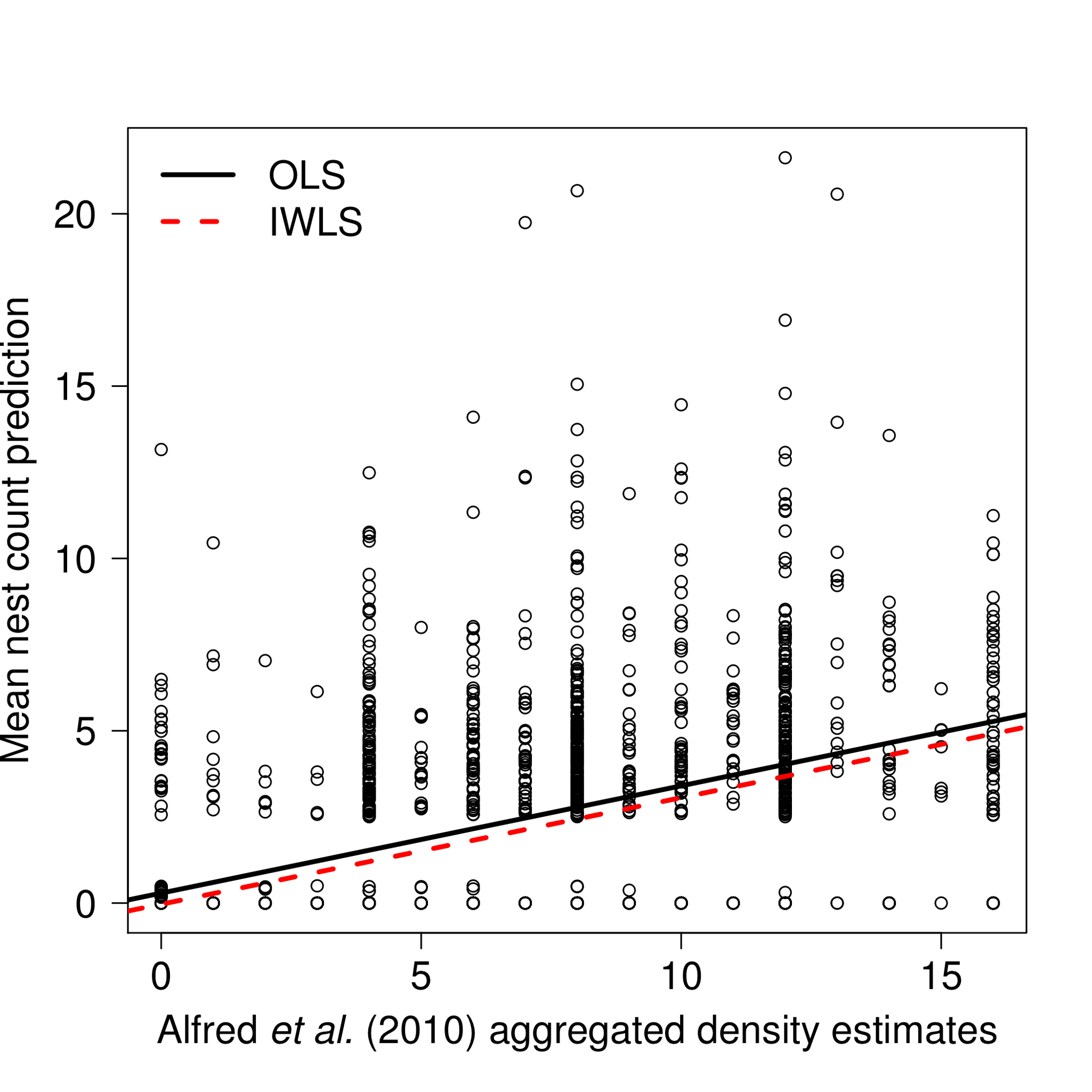

Supplement: Figure S2 — Relationship between nest count predictions and an independent orangutan nest count dataset. Plot showing the ordinary least squares (OLS) and robust iterative re-weighted least squares (IWLS) fits when predicting our SDM model predictions with data published in [25]. (TIFF) [file pone.0043846.s002.tiff]

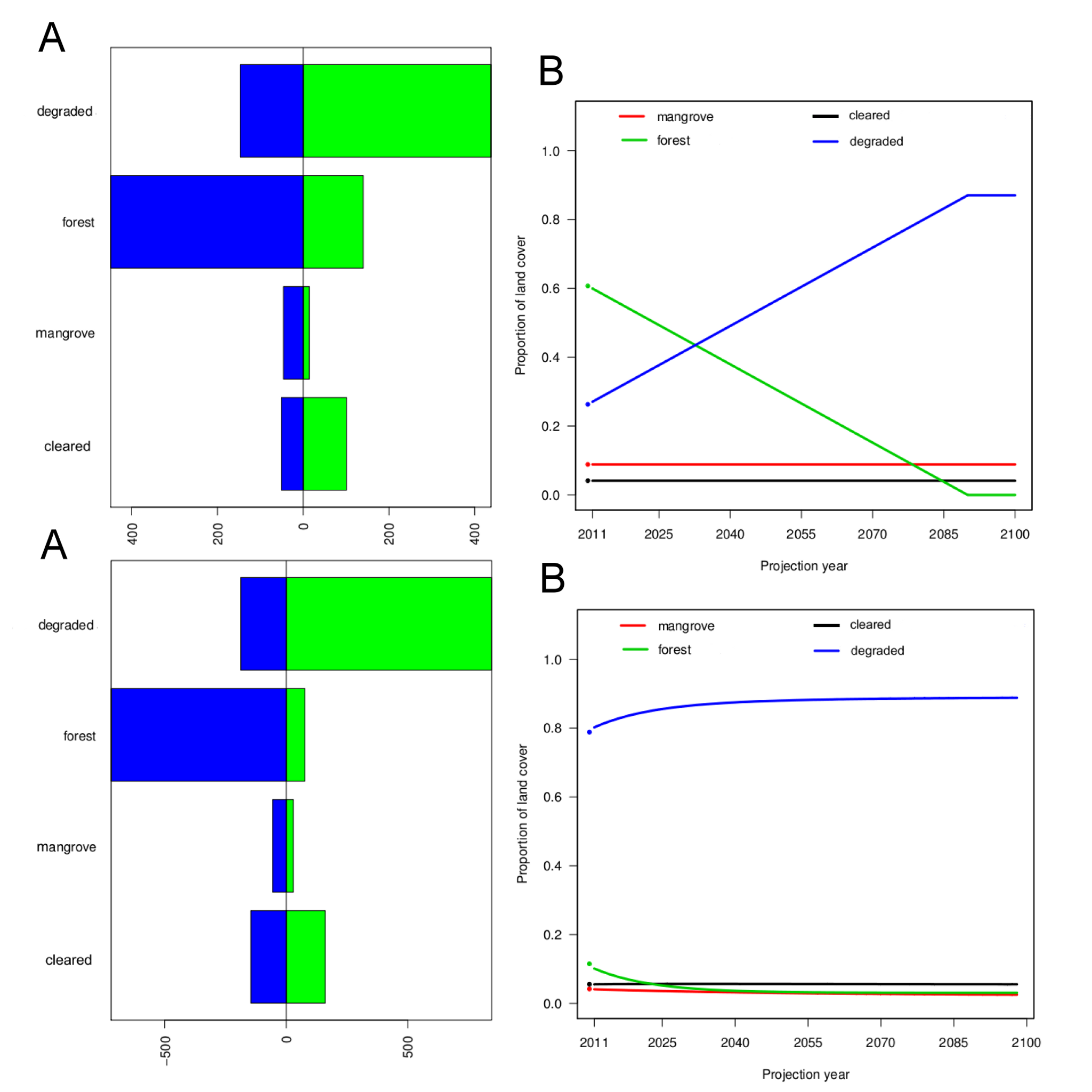

Supplement: Figure S3 — Land-cover class gains and losses and projected changes between 2010–2100. Plots showing (a) the observed gains and losses of cells in each land cover class, and (b) the projected changes in each land class between 2010–2100 in forest reserves and unprotected forests for the current-day SFM scenario. Absolute gains and losses in least-widespread classes were negligible compared to changes in degraded land and forest cover. Consequently, projected changes in these land-cover class were inconsequential compared to projected gains and losses in degraded and forest land cover, respectively. (TIFF) [file pone.0043846.s003.tiff]

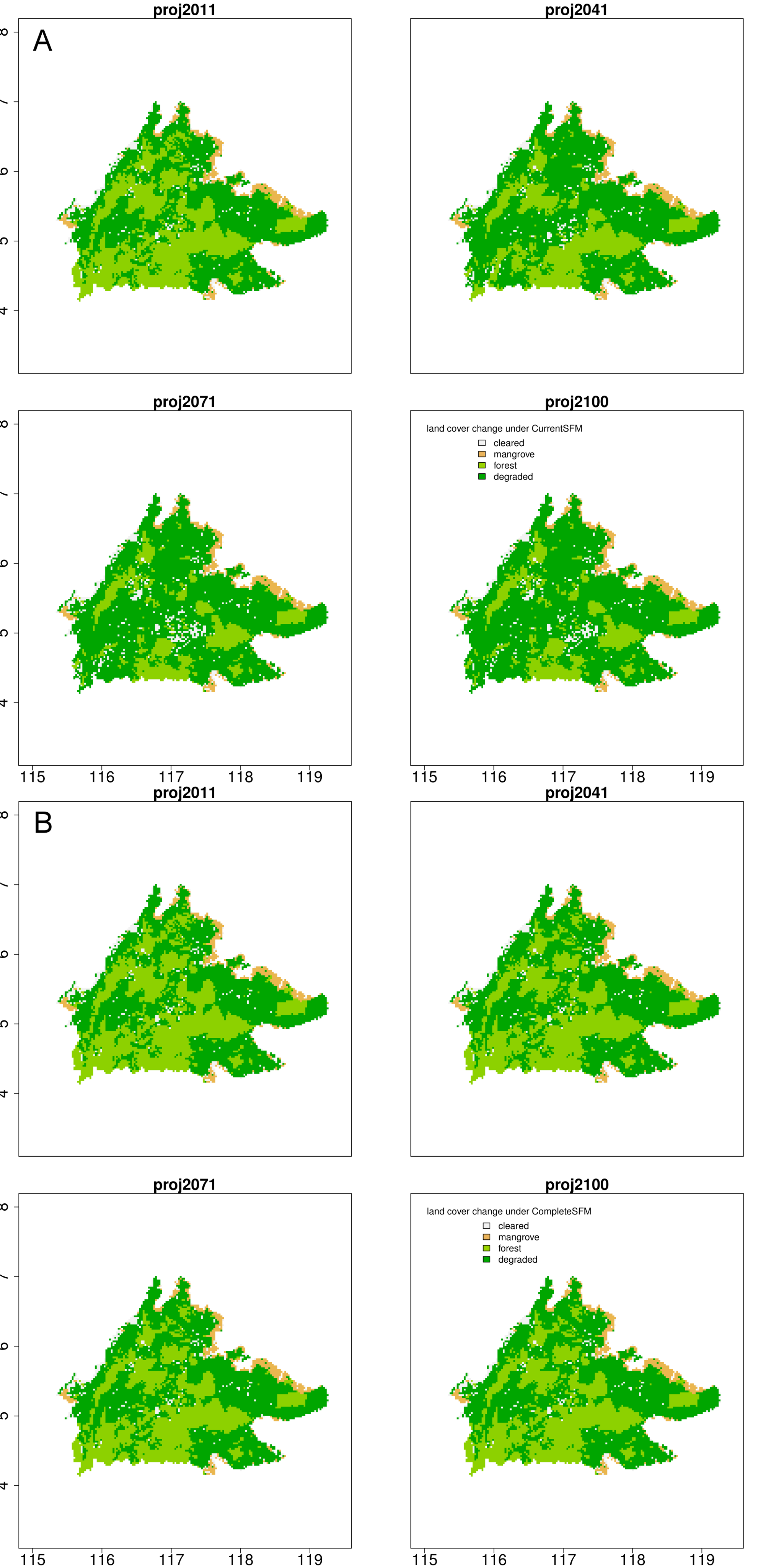

Supplement: Figure S4 — Land-cover change projections. Maps showing land cover change projections at 2011, 2041, 2071 and 2100 under (a) current-day SFM and (b) complete SFM implementations. (TIFF) [file pone.0043846.s004.tiff]

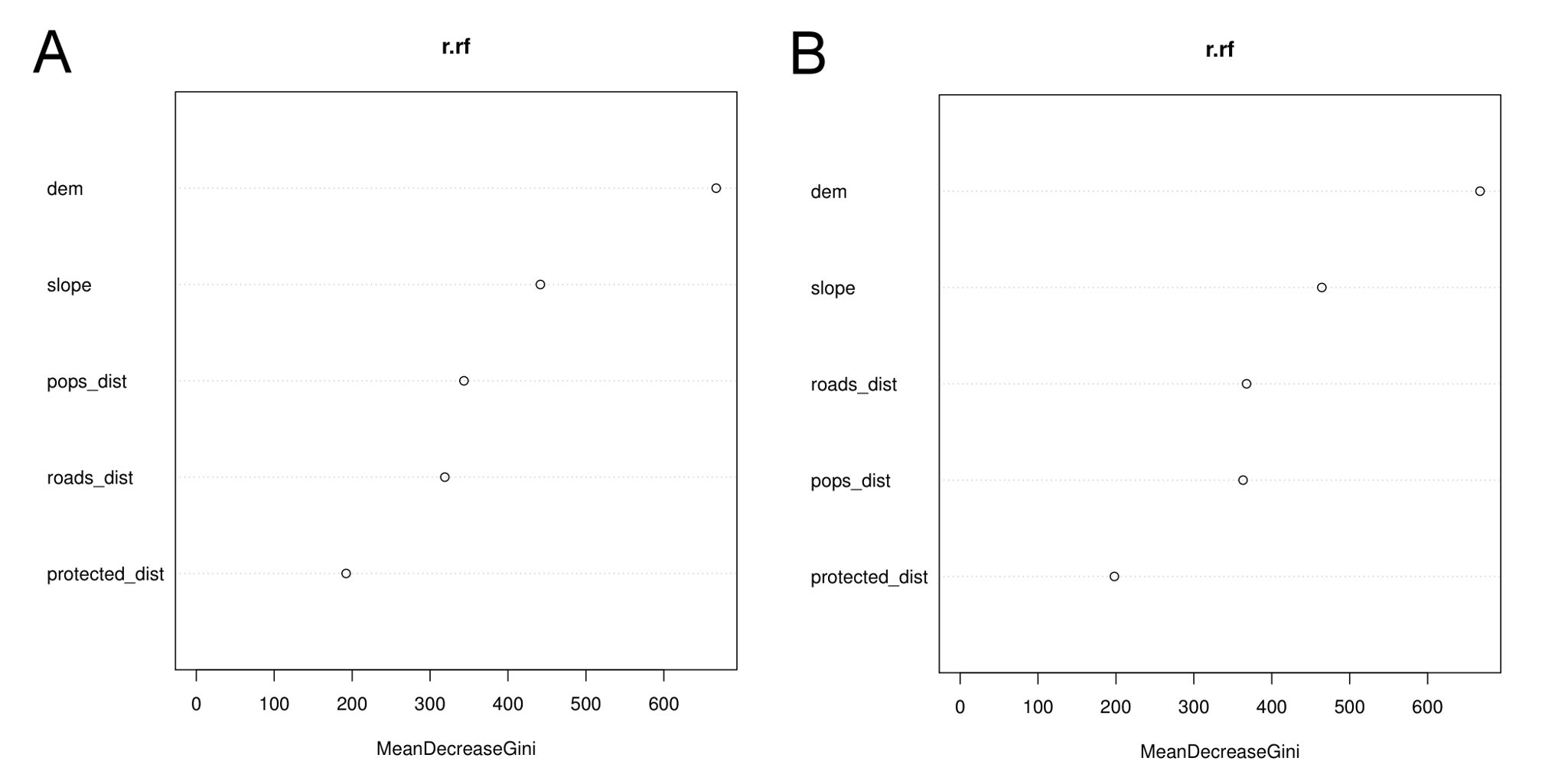

Supplement: Figure S5 — Predictor importance for predicting 2010 observed land-cover. Plots showing the relative importance of spatial predictors in predicting observed 2010 land cover in (a) forest reserves and (b) unprotected forests. (TIFF) [file pone.0043846.s005.tiff]

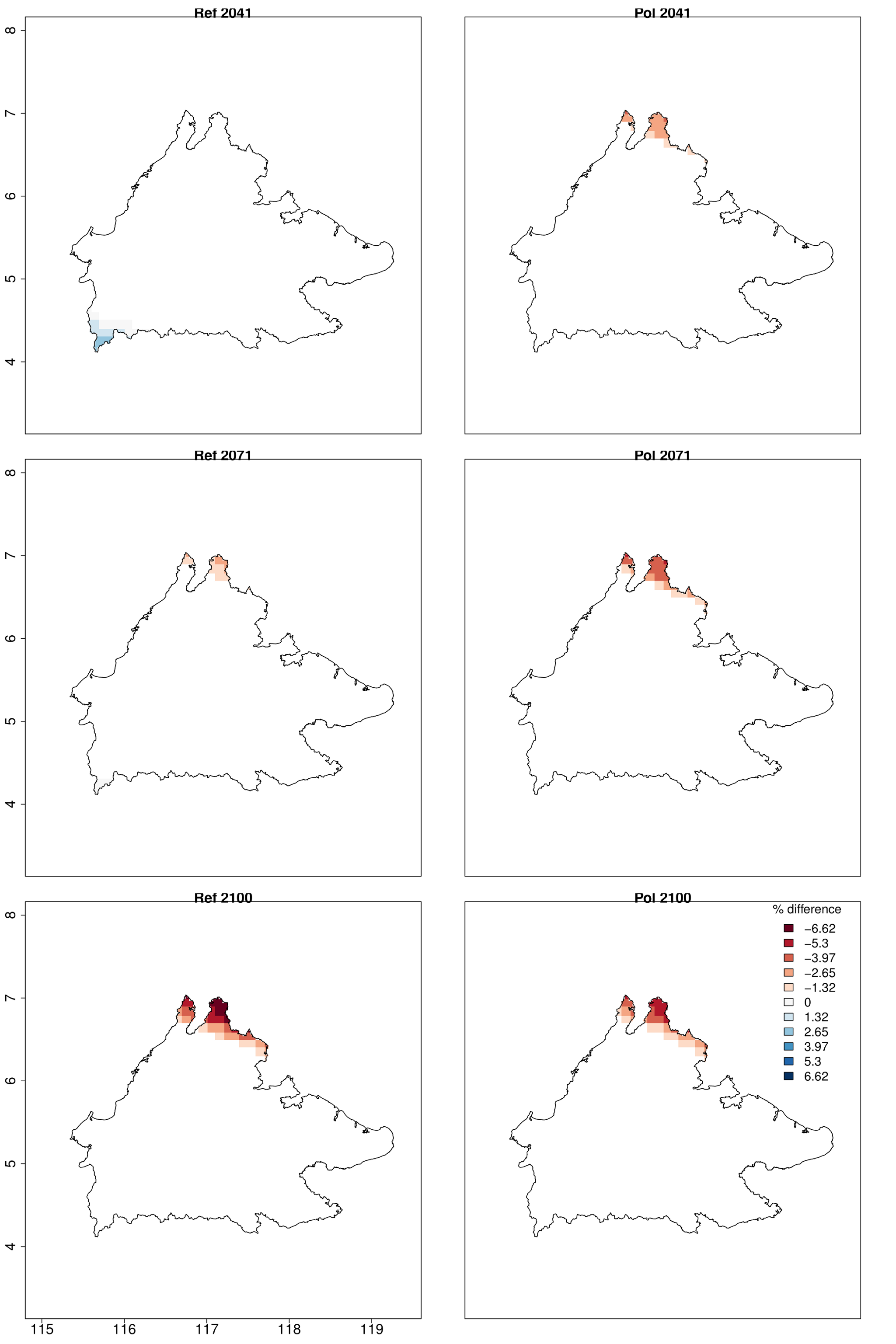

Supplement: Figure S6 — Per cent wet season precipitation delta maps under each CO2 mitigation scenario. Per cent wet season precipitation delta maps at 2041, 2071 and 2100 relative to 2010 for Ref and Pol scenarios. (TIFF) [file pone.0043846.s006.tiff]

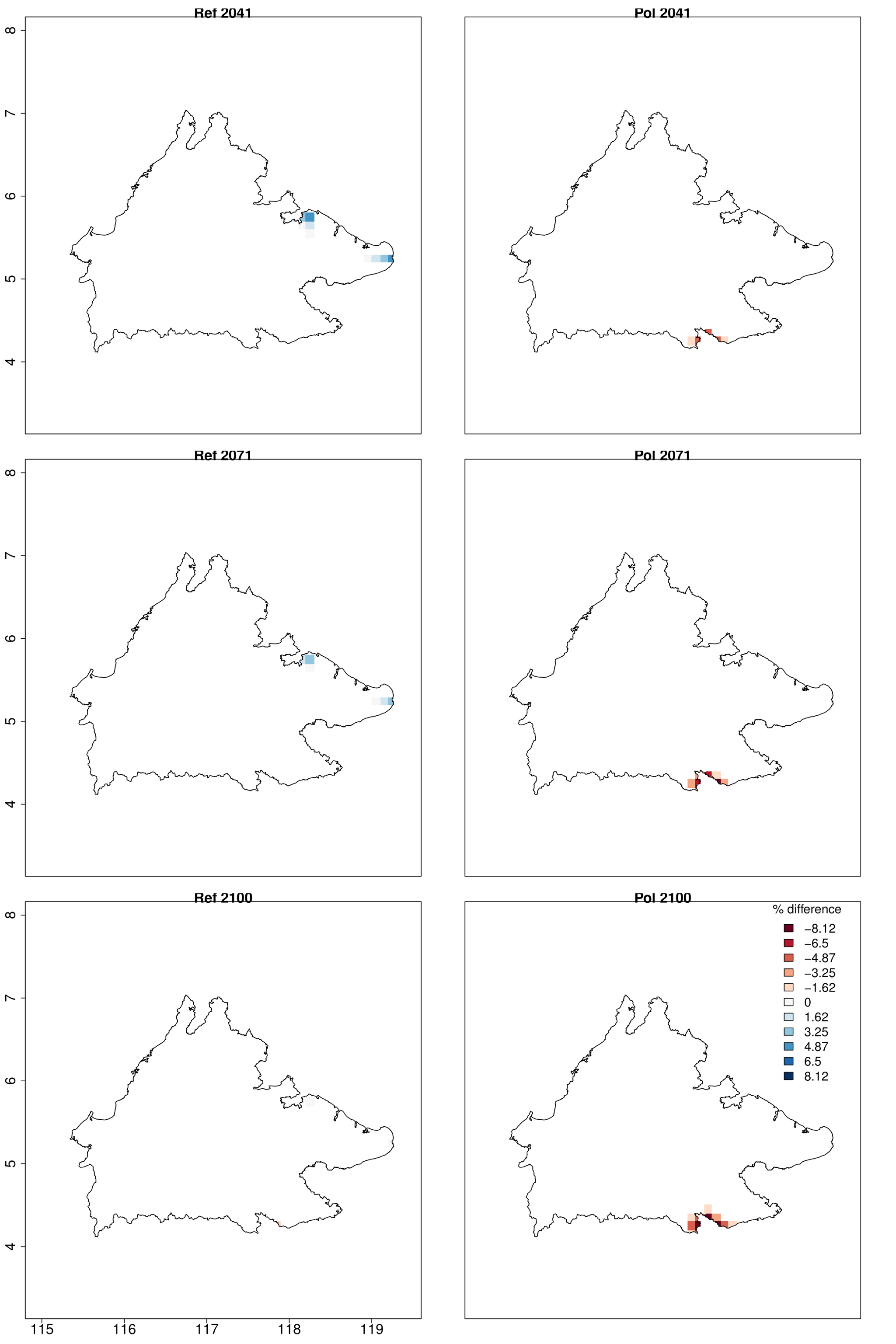

Supplement: Figure S7 — Per cent dry season precipitation delta maps under each CO2 mitigation scenario. Per cent dry season precipitation delta maps at 2041, 2071 and 2100 relative to 2010 for Ref and Pol scenarios. (TIFF) [file pone.0043846.s007.tiff]

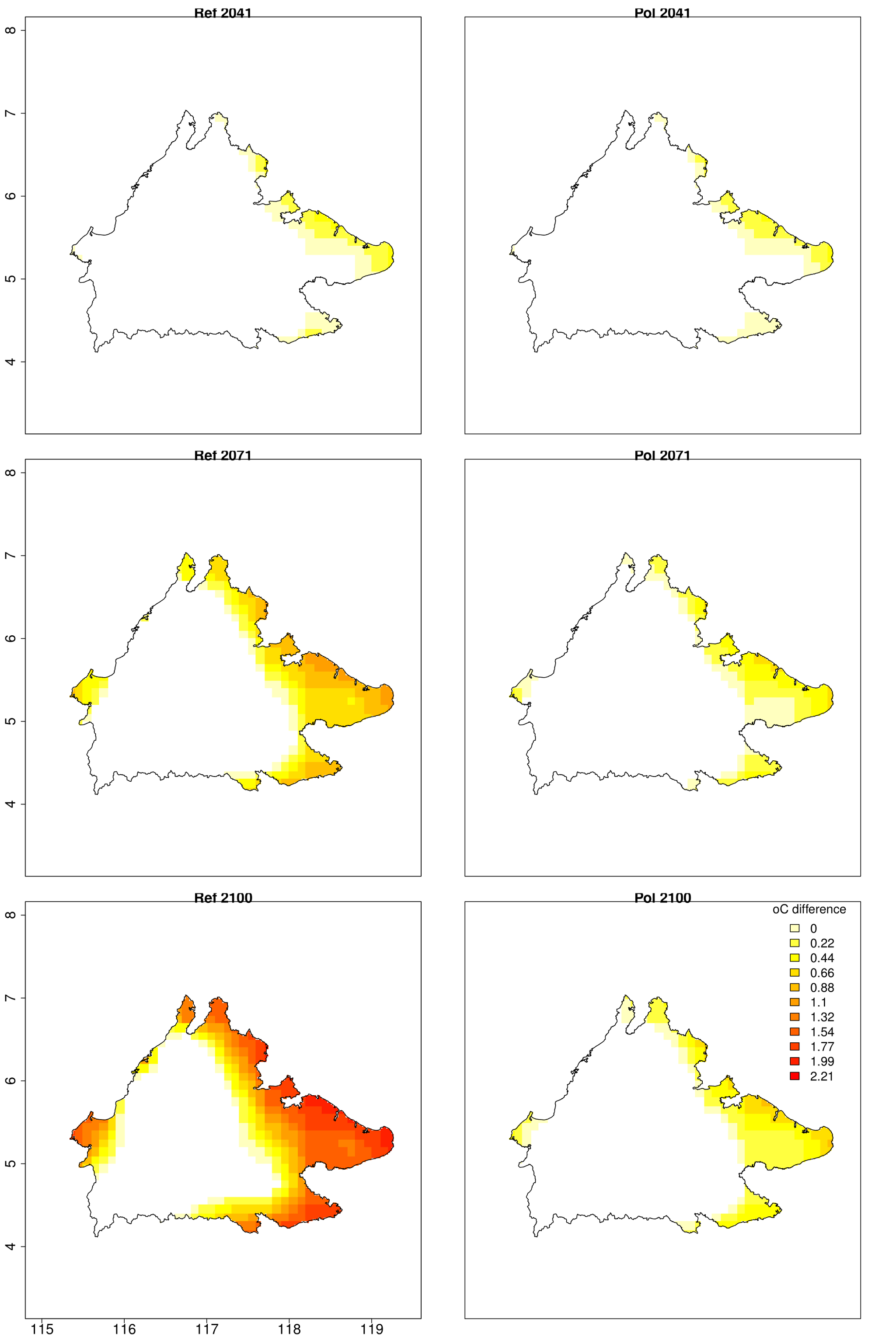

Supplement: Figure S8 — Degree Centigrade temperature delta maps under each CO2 mitigation scenario. Degrees Centigrade temperature delta maps at 2041, 2071 and 2100 relative to 2010 for Ref and Pol scenarios. (TIFF) [file pone.0043846.s008.tiff]
