# Supplementary material for: Long-Term Field Data and Climate-Habitat Models Show That Orangutan Persistence Depends on Effective Forest Management and Greenhouse Gas Mitigation
Source: PLoS One. 2012 Sep 7;7(9):e43846. doi: 10.1371/journal.pone.0043846 (PMC3436794; doi:10.1371/journal.pone.0043846)
Supplement: Table S1 — Spatial predictors. Spatial predictors used to build the Species Distribution Models and notes on their processing. (DOC) [file pone.0043846.s009.doc]

**Table S1. Spatial predictors.** Spatial predictors used to build the Species Distribution Models and notes on their processing.

| Name | Processing notes |
| --- | --- |
| distance rasters | Vector data were rasterized at 0.00895 degree resolution (1 km). Cost distance was calculated assuming a uniform cost surface and allowing Knight’s moves. Resulting raster was upscaled to 0.02237 degree resolution (2.5 km) by the mean. |
| protected raster | Vector data were rasterized at 0.00895 degree resolution (1 km) and upscaled to 0.02237 degree resolution (2.5 km) by the mode. |
| elevation raster | Raster data was upscaled from 0.00225 degree resolution (250 m) to 0.02237 degree resolution (2.5 km) by the mean. |
| slope raster | Slope was calculated from the elevation layer as the degrees of inclination from the horizontal. |
| climate rasters | Monthly Climate Research Unit (CRU) ts3.1 precipitation and mean temperature data were averaged across wet and dry season months between 1989-2009. These data were at 0.5 degree resolution (50 km) and did not overlap the entire land area. The data coverage was expanded by calculating empty edge cells as the average of their nearest 2 cells and downscaled to 10 km using bilinear interpolation. Final data were sampled to 0.02237 degree resolution (2.5 km) using the mean. |
| forest mangrove & degraded rasters | The 2010 CRISP land cover raster was upscaled from 0.00225 degree resolution (250 m) to 0.00895 degree resolution (1 km) by the mode. Forest and degraded rasters were calculated from the upscaled CRISP raster by recoding original categories as forest = peatswamp; lowland; lower and upper montane forests and degraded = plantation/regrowth; lowland and montane mosaic forests. Mangroves were mangroves. Other land cover types - including oil palm plantation - were used in a mask. These rasters were upscaled to 0.02237 degree resolution (2.5 km) using the mode. |
| mask raster | A raster mask with extent north = 7.35; south = 3.95; east = 119.6; and west = 114.8; was calculated from the upscaled CRISP land cover raster by recoding all forest; mangrove and degraded categories to 1 and water (including ocean); lowland and upland open; urban and large-scale oil palm plantation to NULL. Offshore islands were also set to NULL to restrict analysis to mainland Sabah. All NULL areas were considered completely inhospitable to orangutans and were not included in the analyses. |
