# Supplementary material for: Long-Term Field Data and Climate-Habitat Models Show That Orangutan Persistence Depends on Effective Forest Management and Greenhouse Gas Mitigation
Source: PLoS One. 2012 Sep 7;7(9):e43846. doi: 10.1371/journal.pone.0043846 (PMC3436794; doi:10.1371/journal.pone.0043846)
Supplement: Table S2 — Land cover change predictors. Table of variables considered important predictors of land cover change. (DOC) [file pone.0043846.s010.doc]

**Table S2.** **Land cover change predictors.** Table of variables considered important predictors of land cover change.

| Name | Description |
| --- | --- |
| popsdist | distance to a major population centre (250000+ people) |
| roaddist | distance to a main road |
| dem | meters above sea level |
| slope | degrees of inclination from the horizontal |
| protecteddist | distance from protected areas |
