# Supplementary material for: Long-Term Field Data and Climate-Habitat Models Show That Orangutan Persistence Depends on Effective Forest Management and Greenhouse Gas Mitigation
Source: PLoS One. 2012 Sep 7;7(9):e43846. doi: 10.1371/journal.pone.0043846 (PMC3436794; doi:10.1371/journal.pone.0043846)
Supplement: Table S3 — Accuracy statistics for the random forest models of 2010 land-cover predictions. Prediction error rates were low for the most-widespread land-cover classes but high for the least-widespread classes (e.g., mangrove and cleared land) because they constituted less than 5% of the land cover. (DOC) [file pone.0043846.s011.doc]

**Table S3.** **Accuracy statistics for the random forest models of 2010 land-cover predictions.** Prediction error rates were low for the most-widespread land-cover classes but high for the least-widespread classes (e.g., mangrove and cleared land) because they constituted less than 5% of the land cover.

|  |  | Classification error rate | | | |  |  |  |
| --- | --- | --- | --- | --- | --- | --- | --- | --- |
| Forest type | OOB | Cleared | Mangrove | Forest | Degraded | k | Sens | Spec |
| Forest reserve | 22.90% | 0.831 | 0.133 | 0.086 | 0.507 | 0.554 | 0.760 | 0.370 |
| Unprotected forest | 17.42% | 0.807 | 0.563 | 0.609 | 0.047 | 0.405 | 0.641 | 0.492 |

Abbreviations: OOB is Out-Of-Bag error rate, k is kappa, Sens is Sensitivity (true positives) and Spec is Specificity (true negatives).
